# Role of mesenchymal stem cells in osteosarcoma and metabolic reprogramming of tumor cells

**DOI:** 10.18632/oncotarget.2243

**Published:** 2014-07-28

**Authors:** Gloria Bonuccelli, Sofia Avnet, Giulia Grisendi, Manuela Salerno, Donatella Granchi, Massimo Dominici, Katsuyuki Kusuzaki, Nicola Baldini

**Affiliations:** ^1^ Department of Biomedical and Neuromotion Sciences, Alma Mater Studiorum-University of Bologna, Bologna, Italy; ^2^ Laboratory for Orthopedic Pathophysiology and Regenerative Medicine, Rizzoli Orthopedic Institute, Bologna, Italy; ^3^ Department of Medical and Surgical Sciences for Children & Adults, University-Hospital of Modena and Reggio Emilia, Modena, Italy; ^4^ Department of Molecular Cell Physiology, Kyoto Prefectural University of Medicine, Graduate School of Medical Science, Kyoto, Japan

**Keywords:** osteosarcoma, mesenchymal stem cells, lactate, MCT-1, MCT-4

## Abstract

The tumor microenvironment plays an important role in cancer progression. Here, we focused on the role of reactive mesenchymal stem cells (MSC) in osteosarcoma (OS), and used human adipose MSC and a panel of OS cell lines (Saos-2, HOS, and 143B) to investigate the mutual effect of normal-cancer cell metabolic programming. Our results showed that MSC are driven by oxidative stress induced by OS cells to undergo Warburg metabolism, with increased lactate production. Therefore, we analyzed the expression of lactate monocarboxylate transporters. By real time PCR and immunofluorescence, in MSC we detected the expression of MCT-4, the transporter for lactate efflux, whereas MCT-1, responsible for lactate uptake, was expressed in OS cells. In agreement, silencing of MCT-1 by siRNA significantly affected the ATP production in OS cancer cells. Thus, cancer cells directly increase their mitochondrial biogenesis using this energy-rich metabolite that is abundantly provided by MSC as an effect of the altered microenvironmental conditions induced by OS cells. We also showed that lactate produced by MSC promotes the migratory ability of OS cells. These data provide novel information to be exploited for cancer therapies targeting the mutual metabolic reprogramming of cancer cells and their stroma.

## INTRODUCTION

In addition to cancer cells, a number of reactive elements are present in the tumor microenvironment, including endothelial cells, immune cells, and fibroblast-like stromal cells, playing a key role in the establishment and progression of the tumor [1]. In particular, the so-called cancer-associated fibroblasts (CAF) [2, 3], that are thought to originate from mesenchymal stem cells (MSC) [4, 5], have been described as key players in tumor metabolism. According to Otto Warburg's findings, a hallmark of cancer cells is anaerobic glycolysis even under aerobic conditions. This behavior involves increased glucose consumption with consequent excessive production of lactate and H^+^, which causes a decrease of the extracellular pH to levels lower than in normal cells [6]. Indeed, most normal cells have a low rate of glycolysis followed by oxidation of pyruvate in mitochondria. The Warburg effect has important medical applications as high aerobic glycolysis has been clinically confirmed in most of malignancies.

Recent studies have shown that the Warburg effect is also activated in CAF that secrete energy-rich glycolytic metabolites, such as lactate and ketones. Cancer cells are able to uptake these metabolites and use them in the mitochondrial TCA cycle (the so-called reverse Warburg effect). Thus, there is a higher production of energy that therefore drives a large number of the cell's energy-requiring processes. This role of CAF as tumor feeders has been reported in human breast [7, 8], prostate [9], and head and neck cancer [10].

CAF have also been established as an important component of osteosarcoma (OS) [11, 12], a highly malignant tumor of bone that typically affects children and adolescents. OS rapidly invades and destroys the surrounding bone and soft tissues, and in over 90% of the cases spreads to the lung within a few months after diagnosis. Radical surgical removal of the primary followed by aggressive multi-agent chemotherapy has been developed as the treatment of choice of OS, with a significant improvement in survival [13], although less toxic, more active agents are strongly felt to be needed. In this respect, most studies have focused on the molecular features of OS cells.

In this study, we considered the tumor microenvironment of OS as a whole and set out to inquire into the metabolic interactions between the stroma and tumor compartments. We employed OS cell lines such as Saos-2, HOS, and 143B. Our hypothesis is that the cellular metabolism of OS is deeply altered by MSC, affecting their behavior not only in the primary tumor site but also at the metastatic niche. Although the origin of OS is largely unknown, three specific aspects suggest the importance of MSC in the pathogenesis of this tumor. First, this tumor likely arises from transformed stem cells of mesenchymal origin [14]. Secondly, the number of circulating MSC is extremely high in OS patients [15]. The third consideration is that OS are able to recruit these circulating MSC [16]. Interestingly, MSC have been demonstrated to provide a fertile microenvironment for OS cells, promoting tumor progression and the formation of metastasis also through the secretion of signaling molecules [12, 17, 18]. We investigated the interaction between MSC and OS cells and, for the first time, were able to demonstrate the induction of aerobic glycolysis in MSC as a consequence of oxidative stress induced by OS cells. In turn, MSC enhance lactate secretion through an increased expression of MCT-4, and OS cells upload lactate through MCT-1, eventually raising their mitochondrial respiration to facilitate migration.

## RESULTS

### Reciprocal metabolic reprogramming of OS cells and MSC

**Figure 1:**
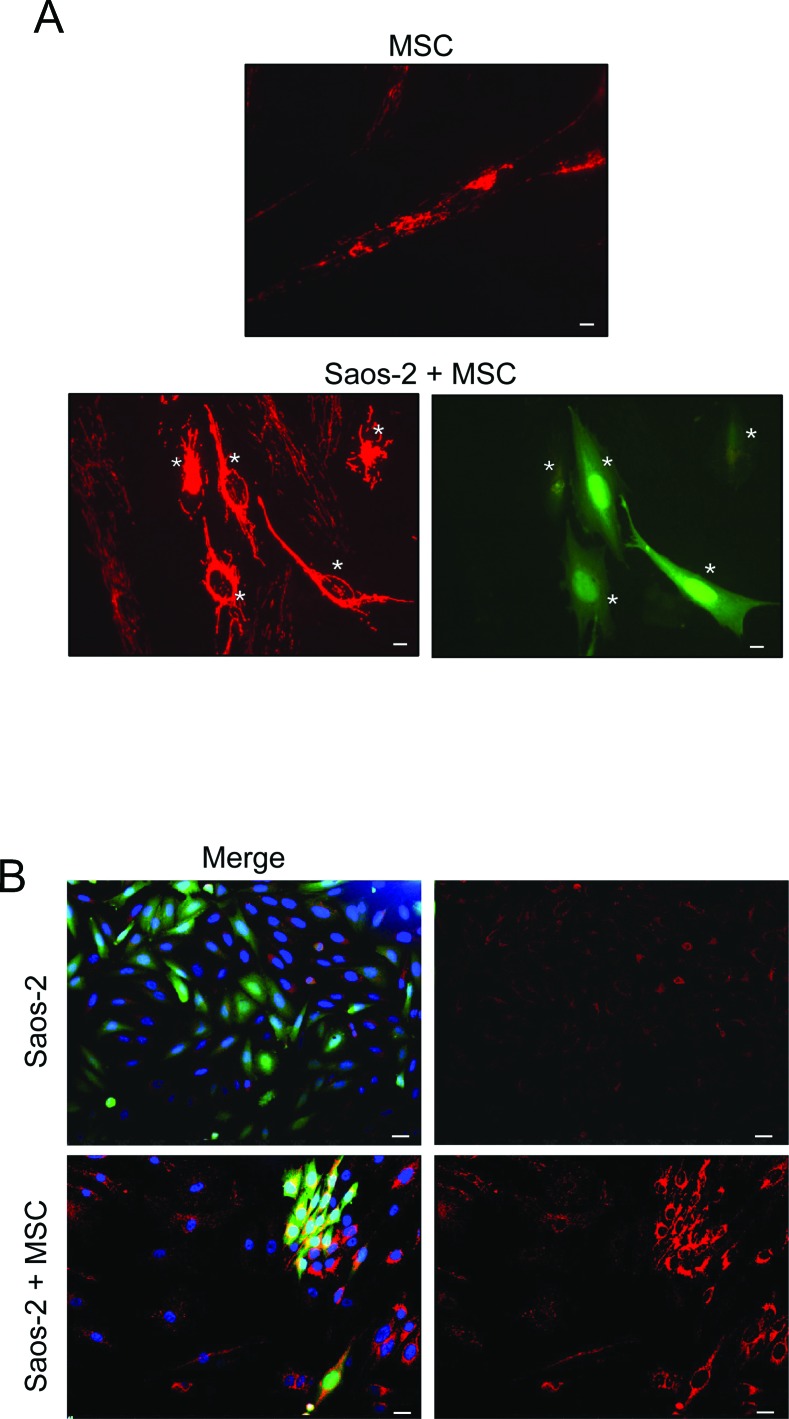
MSC-Saos-2 cell co-cultures exhibit changes in the mitochondrial state **(A)** MSC decrease their mitochondrial activity in co-cultured Saos-2 cells. Co-cultures of Saos-2 GFP (+) positive cells (green) and MSC were immunostained with MitoTracker (red). Note that co-culture with OS cancer cells induces a significant decrease in the mitochondrial activity of MSC (lower row), as compared to MSC cultured alone (top row). Importantly, images were acquired using identical exposure settings. White asterisks indicated Saos-2 GFP (+) positive cells. Original magnification, 20x; scale bar 10 μm. **(B)** MSC induce an increase in mitochondrial mass in co-cultured Saos-2 GFP (+) positive cells (green). Homotypic cultures of Saos-2-GFP (first row, merge first panel) and their co-culture with MSC (lower row, merge first panel) were immunostained with an anti-intact mitochondrial membrane antibody (red). DAPI was used to stain nuclei (blue). Note that in co-culture, the mitochondrial mass is clearly increased in Saos-2 cells as compared to the respective homotypic culture. Importantly, images were acquired using identical exposure settings. Original magnification, 20x. Scale bar 20 μm.

To investigate the bioenergetic status of OS microenvironment, we employed a co-culture system of human OS cells (Saos-2) and human MSC from adipose tissue, and evaluated the mitochondrial activity as compared to homotypic cultures. To this end, Saos-2-MSC co-cultures and corresponding homotypic cultures were first immunostained with MitoTracker, a dye that detects functional mitochondria with active membrane potential. Figure 1A shows that homotypic culture of MSC displays a high mitochondrial activity that is decreased in the co-culture condition, suggesting that the presence of Saos-2 cells induces a mitochondrial dysfunction in MSC.

We next performed immunostaining of OS-MSC co-cultures with an antibody for the intact mitochondrial membrane (MAB1273), to evaluate the mitochondrial mass. Figure 1B shows that MSC increase mitochondrial mass in adjacent Saos-2 GFP (+) cells.

These results indicate that, in the co-culture system mimicking tumor microenvironment, MSC induce mitochondrial activity and biogenesis in OS cells. This increased mitochondrial power may sustain their increased metabolic demands. On the other hand, OS cells decrease mitochondrial power of MSC, inducing their metabolic switch toward an aerobic glycolytic pathway.

### Saos-2 cells induce aerobic glycolysis in MSC

**Figure 2:**
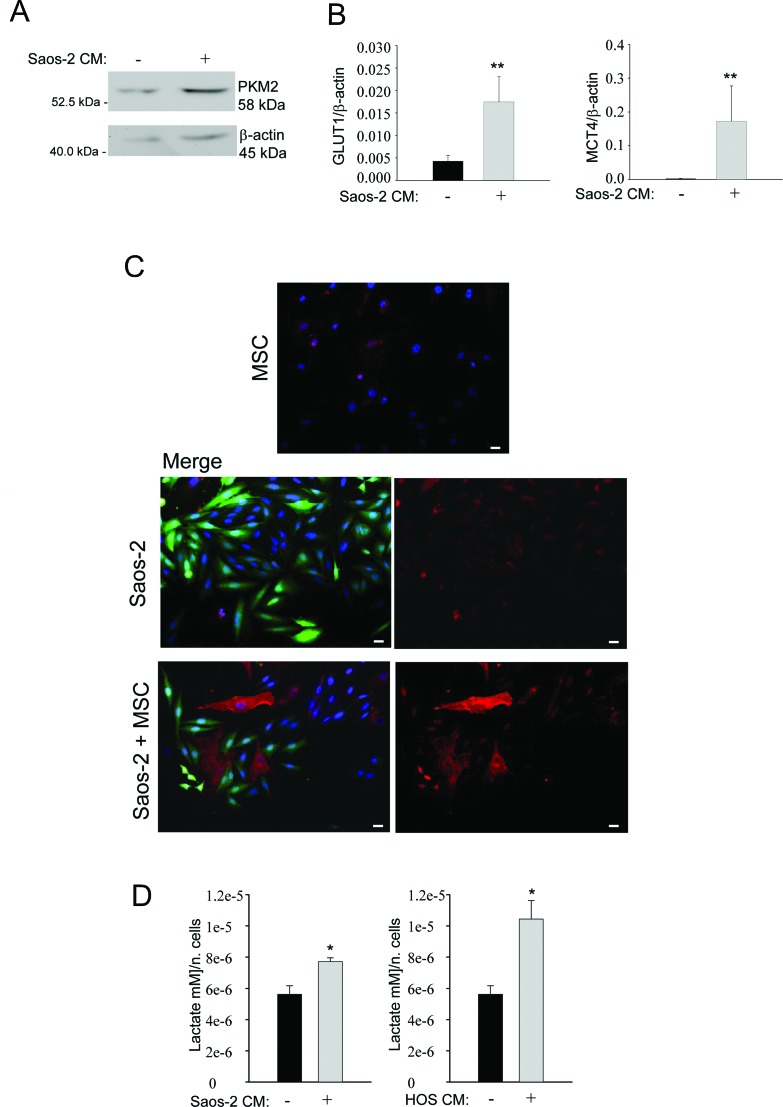
Saos-2 cells drive aerobic glycolysis in adjacent MSC **(A)** Upregulation of PKM2 in MSC activated by OS cells. Cell lysates were prepared from MSC activated by conditioned medium from serum-starved Saos-2 cells or untreated, non-activated MSC. Lysates were analyzed by Western blot with an anti-PKM2 specific antibody. Note the upregulation of PKM2 in tumor-activated MSC, as compared to untreated MSC. Normalization was done by actin immunoblot. **(B)** Gene expression analysis of GLUT1 (left plot) and MCT-4 (right plot) by RT-PCR. Note that MSC cultured with CM from Saos-2 cells, show significantly higher levels of the GLUT1 and MCT-4 genes, versus non-activated MSC. Glut1, p=0.0092; MCT-4, p=0.0071. **(C)** Upregulation of MCT-4 expression was validated by immunofluorescence analysis. Homotypic cultures of MSC (top row) and Saos-2 GFP cells (middle row) and Saos-2-MSC heterotypic cultures (lower row) were immunostained with MCT-4 antibodies. DAPI was used to stain nuclei (blue). Note that the MCT-4 expression (red) is clearly increased in MSC in co-culture condition, as compared to MSC cultured alone. Importantly, images were acquired using identical exposure settings. Original magnification, 20x. Scale bar 40 μm. **(D)** Tumor-activated MSC show increased lactate secretion. MSC were incubated with CM obtained from OS cells (Saos-2 or HOS) for 48 hours. Then, cells were carefully washed and incubated in fresh serum-free medium for an additional 24 hours. Lactate assay was performed on this culture medium. Note that lactate production is significantly increased in MSC, after activation with conditioned media from Saos-2 cells (left plot) and HOS cells (right plot). Values were normalized by cell numbers *p<0.05.

Next, we set out to investigate the glycolytic profile of MSC. To this end, we studied the expression of pyruvate kinase isoform 2 (PKM2), a key regulator of glycolysis and promoter of tumor growth [19]. For this purpose, MSC were incubated with conditioned medium (CM) derived from Saos-2 cells for 24 hours. Western blot analysis demonstrates that MSC activated by Saos-2-CM have a higher PKM2 expression, as compared to untreated MSC (Figure 2A), suggesting that the conditioned medium from Saos-2 cells induces a glycolytic switch in MSC. A shift towards a glycolytic phenotype is also supported by the real time PCR analysis for the glucose transporter GLUT1 mRNA in CM-activated MSC versus not activated MSC. Figure 2B (left plot) shows increased GLUT1 expression in MSC after activation with Saos-2-CM, suggesting an enhanced glucose uptake and increased glycolysis.

Next, we investigated the expression of MCT-4, the passive lactate-proton shuttle responsible for the lactate efflux. We found that the CM-treatment increased the gene expression of MCT-4 (Figure 2B, right plot). In agreement with these data, immunofluorescence analysis demonstrates that MSC in co-culture with OS cells show higher MCT-4 expression, as compared to the corresponding homotypic culture (Figure 2C). Given that MSC undergo a glycolytic switch following their activation by OS cells, we next investigated the secretion of lactate, the final product of glycolysis. Importantly, we observed that MSC, after activation with CM from 2 OS cell lines (Saos-2 and HOS cells), show an increased secretion of lactate, with respect to untreated MSC (Figure 2D).

### OS cells uptake lactate produced by activated MSC

**Figure 3:**
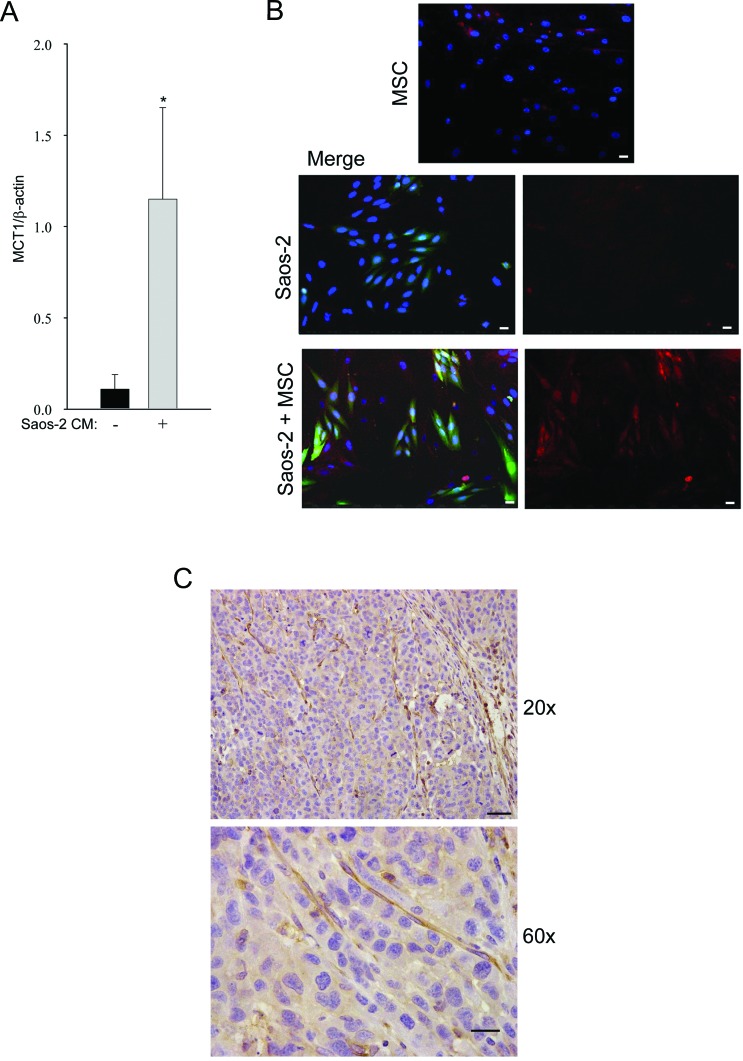
Tumor-activated MSC induce high MCT-1 expression in Saos-2 cells **(A)** The result is observed by RT-PCR, *p<0.05. **(B)** MCT-1 is upregulated in Saos-2-GFP cells co-cultured with MSC. MCT-1 immunofluorescence was performed in homotypic cultures of MSC (top row) and Saos-2 cells (middle row), and in the MSC-Saos-2 cell co-culture system (lower row). Note that MCT-1 expression (red staining) is mainly present in the OS green cells. Merged image is shown in the middle and lower panels. DAPI was used to stain nuclei (blue). Importantly, images were acquired using identical exposure settings. Original magnification, 20x. Scale bar 40 μm. **(C)** MCT-4 immunohistochemistry was performed on mouse tumor xenograft of human 143B cells. Note that tumor stroma from the host is strongly stained for MCT-4 (brown), while the tumor compartment is poorly stained. Original magnification 20x; scale bar 50 μm (upper row). Original magnification 60x and scale bar 20 μm (lower row).

We next investigated the expression of MCT-1, the monocarboxylate transporter for the uptake of lactate, in OS cells, by real time PCR. Saos-2 cells, after incubation with CM of activated MSC, significantly increased the expression of MCT-1, as compared to untreated cells (Figure 3A).

These results were confirmed by immunofluorescence, showing MCT-1 upregulation in OS cells co-cultured with MSC, as compared to OS cells cultured alone (Figure 3B). These results suggest that OS cells are able to uptake lactate, a high-energy metabolite which is a substrate for mitochondrial oxidative phosphorylation.

To assess if the metabolic reprogramming observed *in vitro* also occurs *in vivo*, 143B human OS cells were injected into the flanks of SCID mice. After three weeks, tumors were collected and immunohistochemistry was performed on paraffin sections for the expression of MCT-4, the transporter for lactate extrusion. As shown in Figure 3C, MCT-4 was highly expressed in the stromal compartment of the xenograft tumors. These findings indicate that, also *in vivo,* glycolysis preferentially occurs in the stromal compartment, leading to increased MCT-4 expression. This suggests that stromal cells avoid the internal accumulation of lactate, making it available for OS cells. Thus, our data indicate that tumor cells induce important metabolic alterations in adjacent stromal cells, with impairment of their mitochondrial function and enhancement of aerobic glycolysis.

### Aerobic glycolysis in MSC is ROS-dependent

**Figure 4:**
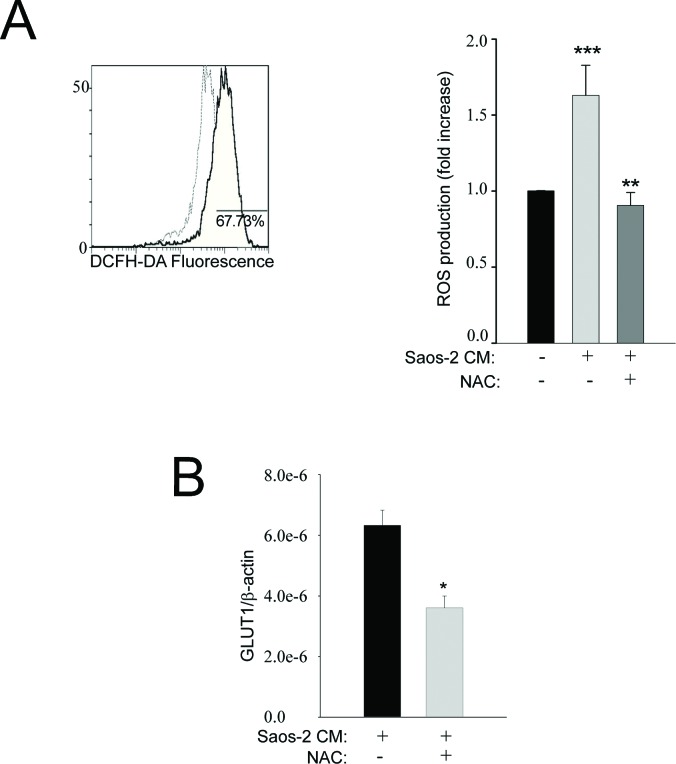
Oxidative stress is increased in activated MSC cells **(A)** MSC cells were exposed to conditioned medium from OS cells and evaluated by flow cytometry using DCFH-DA (as a measure of total ROS released). The representative plot of the flow-cytometric analysis shows that about 70% of MSC exposed to the Saos-2 cells-conditioned medium (continuous dark line) exhibit a fluorescence intensity higher than that observed in untreated MSC (dashed light line). The histogram bars show the ratio between “MC of activated MSCs” and “MC of non-activated MSC” (mean ± SEM). The ROS production in MSC challenged with the conditioned medium of Saos-2 cells was significantly increased in comparison to untreated MSC, but the basal activity of MSC is restored when an antioxidant, i.e. NAC, is introduced into the culture system. **p= 0.002; ***p= 0.0005. MC: mean channel of fluorescence intensity. **(B)** Pre-treatment of MSC with the anti-oxidant NAC decreases the expression of GLUT-1, suggesting that the shift to the Warburg metabolism with increased uptake of glucose depends on oxidative stress, *p<0.05.

Oxidative stress is known to drive tumor spread and invasion [20, 21] and this phenomenon has already been shown in stromal fibroblasts from breast and prostate cancer and suggested as a starter of glycolytic switch [9, 22]. Figure 4A (representative plot) shows that over 70% of MSC cells exposed to OS-conditioned medium have higher levels of ROS, with respect to non-activated MSC. Interestingly, the basal ROS levels of MSC were restored when cells were treated with the antioxidant N-Acetyl-Cystein (NAC) (Figure 4A, graph bar). Accordingly, the expression of the glucose transporter GLUT1 was also decreased in the presence of NAC (Figure 4B). These findings indicate that MSC undergo aerobic glycolysis as a consequence of a ROS-dependent interplay with OS cancer cells.

### Lactate promotes mitochondrial biogenesis and oxidative phosphorylation in Saos-2 cells

**Figure 5:**
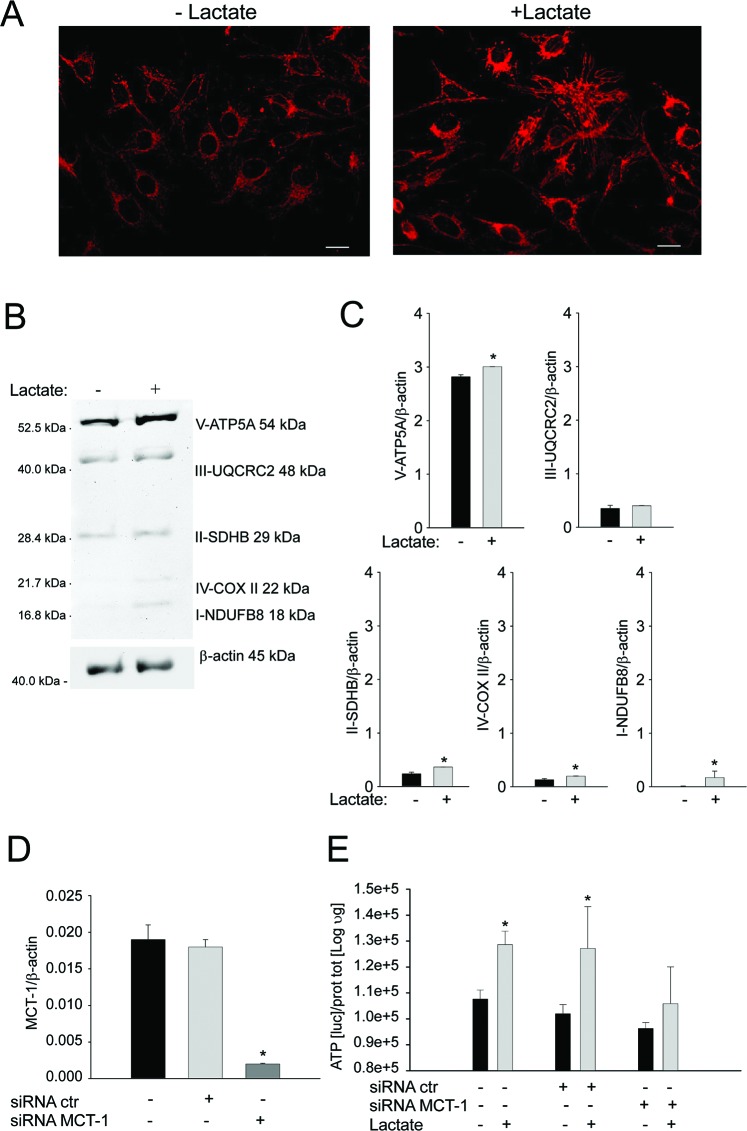
Lactate treatment promotes mitochondrial biogenesis and oxidative phosphorylation in OS cells **(A)** Homotypic cultures of Saos-2 cells were treated with or without 10 mM of lactate for 48 hours. Cells were fixed and immunostained with an antibody against the mitochondrial membrane (red). Note that lactate treatment increases mitochondrial mass in Saos-2 cells, mimicking the co-culture condition. Importantly, images were acquired using the same exposure settings. Original magnification, 20x; scale bar 50 μm. **(B)** Immunoblot analysis of V-ATP5A, III-UQCRC2, II-SDHB, IV-COXII and I-NDUFB8 was performed on Saos-2 cells with or without 10 mM of lactate for 48 hours. Actin immunoblot was used for normalization. **(C)** The plot reports the densitometric quantitation (ratio of each protein:actin). Note that complex I, complex II, complex IV and V are upregulated in Saos-2 cells after treatment with lactate, as compared to the untreated samples. *p<0.05. **(D)** Saos-2 cells were treated with MCT-1 specific siRNA and, after 24 hours, the MCT-1 mRNA expression levels were evaluated. The silencing significantly inhibited MCT-1 expression, *p<0.05 vs not treated cells. **(E)** Homotypic cultures of Saos-2 cells transfected with MCT-1 siRNA, with not-targeting siRNA, or untreated, were incubated with or without 10 mM of lactate for 48 hours. Then, ATP content was evaluated. The silencing of MCT-1 expression impaired the increase in ATP content induced by lactate treatment (*p<0.05 treated with lactate vs untreated).

Next, we evaluated if lactate is sufficient per se to induce the effects observed in the co-culture system, i.e. the promotion of mitochondrial biogenesis. To this end, we treated homotypic cultures of Saos-2 cells with 10 mM lactate for 48 hours. After treatment, cells were fixed and immunostained with an antibody against the intact mitochondrial membrane (MAB1273). As shown in Figure 5A, lactate administration strongly increases the mitochondrial mass of OS cells. In addition, we performed Western blot analysis with a panel of antibodies against OXPHOS complex subunits. These subunits must be properly assembled to allow a functional oxidative phosphorylation. As shown in Figures 5B and 5C, upon lactate treatment, OS cells show a strong increased expression of complexes I, II, IV, and V.

Finally, we evaluated if the uptake of lactate that is used by Saos-2 cells for mitochondrial biogenesis, is mediated by MCT-1. For this purpose, we silenced MCT-1 expression in Saos-2 cells by a specific siRNA. Once we confirmed the significant inhibition of MCT-1 mRNA after transfection (Figure 5D), we observed that MCT-1 silencing strongly affected the ATP content of Saos-2 cells treated with lactate. Figure 5E shows that Saos-2 cells treated with an unspecific siRNA or untreated display a significant increase in ATP content after incubation with lactate. Conversely, no significant increase was observed in Saos-2 cells treated with MCT-1 specific siRNA (Figure 5E). These results suggest that OS cells are able to uptake lactate through MCT-1 and utilize this metabolite for their Krebs cycle and ATP synthesis, therefore increasing their bioenergetic status.

### Lactate increases the migratory ability of OS cells

**Figure 6:**
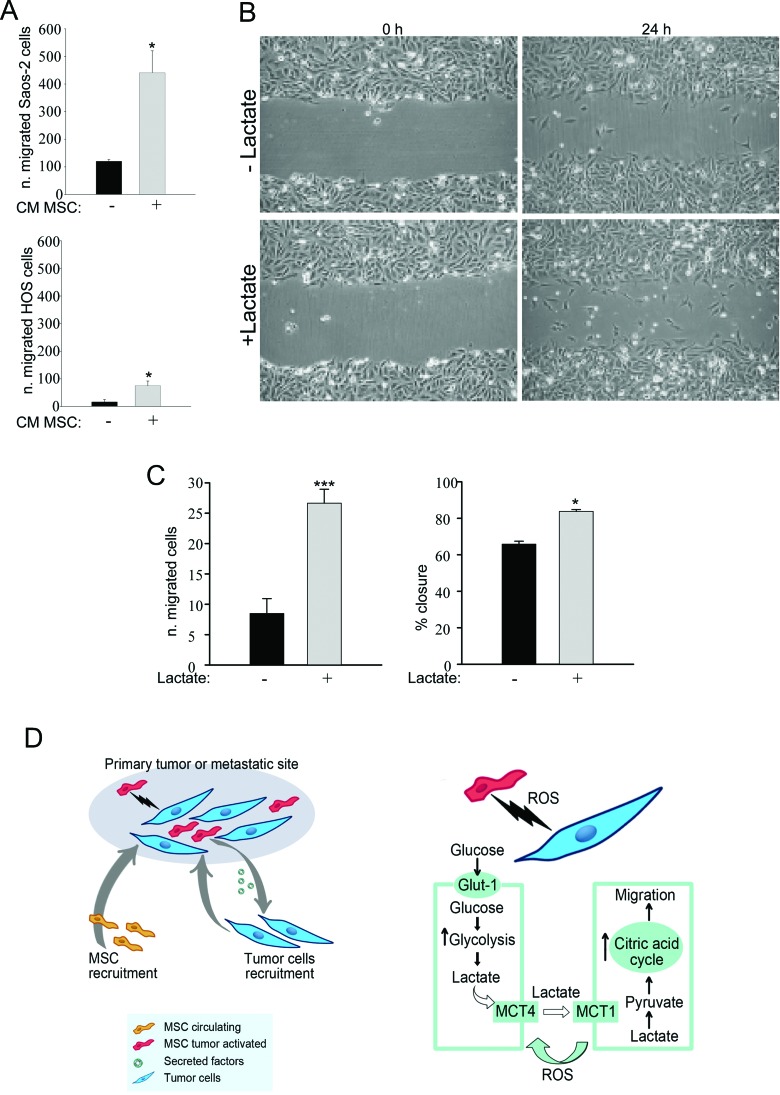
MSC-activated Saos-2 and HOS cells acquire increased migratory capacity **(A)** We assessed whether tumor-activated MSC were able to increase the migration of Saos-2 and HOS cells, using a modified Boyden chamber assay. As a control, serum-free CM from non-activated MSC was placed in the lower chambers. Note that CM of previously activated MSC was able to increase the migratory capacity of OS cells, *p<0.05. **(B** and **C)** We analyzed Saos-2 and HOS migration by an in vitro scratch assay. OS cells were plated to create a confluent monolayer, which was later scraped. Images were acquired with a phase-contrast microscope. To capture the same field during the image acquisition, marks next to the scratch were used as reference points. **(B)** Saos-2 cells were treated with or without 10 mM lactate, as indicated. Images were acquired at 0 and 24 hours. Note that Saos-2 treated with lactate migrate faster, as compared to untreated Saos-2 cells. Original magnification, 10x. **(C)** Quantification of cell migration after scratch assay of Saos-2 cells (left plot) and of HOS cells calculated as % closure (right plot). **(D)** Proposed model of the metabolic circuit between MSC and OS cells. The left drawing represents the primary tumor or the metastatic site that includes tumor cells and MSC as stromal compartment. The right drawing explains in detail the reciprocal metabolic reprogramming: tumor cells induce MSC to undergo Warburg metabolism leading to increased uptake of glucose and its conversion to lactate. Stromal cells extrude lactate through the shuttle MCT-4. OS cells are able to uptake this metabolite through the MCT-1 transporter, and utilizing it as fuel for their TCA cycle, as well as for cell migration.

We then examined the effects of activated MSC on the migratory ability of Saos-2 and HOS cells. For this purpose, MSC were activated by treatment with OS cell-derived conditioned media. Then, to prepare conditioned media from activated MSC, MSC were rinsed and incubated for 24 hours in serum-free media. OS cells were treated with conditioned medium from MSC previously activated, and their migratory ability was assessed using a modified Boyden chamber. We observed that CM from activated MSC significantly enhances cancer cells migration relative to non-activated cells (Figure 6A). Next, we performed an in vitro scratch assay (Figure 6B, representative image) of Saos-2 and HOS cells treated with lactate 10 mM for 24 hours. Lactate treatment significantly increased the migration of OS cells with respect to untreated cells (Figure 6C). These data indicate that MSC are able to promote the migration of OS cells, and this effect is mimicked by lactate treatment (Figure 6D).

## DISCUSSION

The tumor microenvironment is a cellular matrix consisting of cells, soluble factors, signaling molecules and extracellular matrix that supports tumor growth and invasion, protects the tumors from host immunity, fosters therapeutic resistance, and provides a niche for dormant metastases to thrive. As our understanding of the role of the tumor microenvironment in cancer continues to evolve, the complexity of the interactions between cancer cells and their microenvironment has become increasingly apparent. Tumor microenvironment is a dynamic milieu: non-immune mesenchymal cells, such as fibroblasts and their precursors MSC, play an important role in tumor microenvironment where they are “educated” by neoplastic cells and, vice versa, influence the behavior of cancer elements. Controversial results have been reported when MSC injected into mice resulted either in promotion [23, 24, 25] or inhibition [26, 27] of tumor growth. Regarding OS, it has been reported that MSC exert a pro-inflammatory effect on cancer cells by producing soluble factors that promote cell proliferation [4, 28]. However, the effect of MSC within tumors seems unpredictable and dependent on microenvironment signals [29].

In this study, we considered two metabolic compartments in OS, cancer cells and reactive MSC. Our results suggest a model of metabolic coupling by which MSC, after having been “activated” by the OS cells, increase the aggressive behavior of cancer cells. In particular, we showed that tumor cells induce oxidative stress, via ROS production, in adjacent MSC, triggering a metabolic shift to aerobic glycolysis. Moreover, we also showed that MSC in contact with OS cells undergo activation and produce lactate, as described in the Warburg effect. On the other hand, cancer cells increase their mitochondrial activity and their mitochondrial mass by utilizing lactate by an increased MCT-1 expression. This pathological process involving MCT-1 mimics the physiological metabolic situation observed in skeletal muscle fibers, previously described [30]. Our data indicate that the inhibition of MCT-1 significantly affects the energy metabolism of cancer cells, by decreasing ATP production. In agreement, the importance of MCT-1 in OS has been investigated in a recent study, showing that downregulation of MCT-1 inhibits tumor growth and metastasis, and enhances chemotherapeutic efficacy [31].

Moreover, we also found *in vivo* evidence for mitochondrial dysfunction in the stromal compartment of OS mouse tumor xenografts. Indeed, the immunohistochemistry analysis performed on murine tumor sections revealed that MCT-4 was detected only in the stromal host-derived cells. These results suggest not only that OS cells are able to recruit reactive stromal elements, but also that these cells can be induced to undergo aerobic glycolysis and fuel the tumor metabolic needs, in line with results previously observed in breast and head and neck cancer [10, 32]. In addition, we further demonstrated that activated-MSC induce tumor migration and that lactate is directly responsible for such increased migratory capacity of cancer cells. A previous study had shown that MSC drive proliferation and metastasis in OS through IL-6/STAT3 signaling pathway [18]. It remains to be determined if the STAT3 pathway plays a role in our model.

Here, for the first time, we have addressed the role of MSC as modulators of OS metabolism and behavior (Figure 6D). In fact, we demonstrated that MSC play a role as tumor cells feeders after their metabolic reprogramming to a glycolytic phenotype (reverse Warburg phenotype), while, at the same time, tumor cells undergo mitochondrial biogenesis and increased mitochondrial activity. The importance of the increased mitochondrial mass and bioenergetics in tumorigenesis has been also confirmed by recent findings regarding the mitochondrial citrate transporter CIC/ SLC25A1 [45, 46]. CIC is necessary for the Krebs cycle and oxidative phosphorylation. Interestingly, the authors demonstrated that the genetic CIC inhibition induced the Warburg effect in cancer cells, and inhibited tumor growth.

Several studies have shown the importance of resident mesenchymal reactive cells in supporting tumor progression and metastasis in prostate, breast [33], ovarian and colon cancer [34, 35]. It has been recently shown in an animal model that the administration of exogenous MSC may promote tumor engraftment and lung colonization, leading to OS growth and metastasis [12]. These findings, together with the evidence of an increased number of circulating MSC observed in OS patients, and the interesting observation that MSC are capable to facilitate cancer migration through heterotypic interactions with tumor cells [36, 37] [12], suggest that MSC may be important for metastases development, by providing a supportive niche in certain organs. Additionally, the emerging cancer stem cell hypothesis has complicated the scenario. Indeed, it has been reported the presence of stem-like cells, implicated in tumorigenesis, metastasis and drug resistance [38, 39]. Speculatively, MSC may also exist in the cancer stem cell niche, perhaps playing a key role in the subsistence of the microenvironment. Here, we propose a cellular model applicable to the primary tumor site and to the metastatic environment.

Although these data clarify some aspects of the interplay between MSC and tumor cells in aerobic condition, at the same time they bring intricacy to the system. As solid tumors have more hypoxic and acidic microenvironments than normal tissue [40], further investigations should be extended to MSC metabolic behaviors under hypoxic condition and at different pH conditions.

In conclusion, given that osteosarcomas are rare tumors with poor prognosis because of metastatic dissemination, it is of vital importance to understand the interactions between MSC and their niche to possibly find therapeutic strategies.

## METHODS

### Cell cultures and stable transfection

Human mesenchymal stem cells were purchased from ATCC (ATCC^®^ PCS-500-011™) and characterized as described [41, 42]. The OS cell lines Saos-2 (ATCC^®^ HTB-85™) and HOS (ATCC^®^ CRL-1543™) were from ATCC, and the cell line 143B was a kind gift from Dr. Michiel Pegtel (VU University Medical Center, Amsterdam, NL). All cells were maintained in RPMI with 10% fetal bovine serum (FBS) and penicillin 100 units/ml-streptomycin 100 μg/ml. For co-culture experiments, MSC and Saos-2 were plated on glass cover slips in 12-wells plates in 1 ml of complete medium. OS cells were plated within 2 hours of MSC plating. Experiments were performed at a 3:1 MSC-to-OS cell ratio. As controls, monocultures of MSC and OS cells were seeded using the same number of cells as the corresponding co-cultures. The day after plating, medium was changed to RPMI with 10% NuSerum (BD Biosciences) a low protein alternative to FBS, and Pen-Strep. Cells were maintained at 37°C in a humidified atmosphere containing 5% CO_2_. To obtain activated MSC, cells were grown to 80% confluence and treated for 24 hours with conditioned medium from Saos-2 cells in serum-free medium for 48 hours. Activated Saos-2 cells were obtained by incubating them for 24 hours with conditioned medium of MSC previously activated by tumor cells. Saos-2 cells were used to generate a cell line overexpressing the GFP tag. A plasmid purchased from Addgene was employed, and cells were transfected with Fugene6 transfection reagent (Roche) following the manufacturer's instructions. Transfected OS cells were selected with G418/Geneticin (Life Technologies).

### Mitochondrial staining

To evaluate the mitochondrial activity, cells were stained with MitoTracker Orange CMTMRos (M7510) (Invitrogen). Lyophilized MitoTracker was dissolved in DMSO to generate a 1 mM stock solution that was diluted into serum-free DMEM at a final concentration of 25 nM. Briefly, cells were cultured for 48 hours and then stained with MitoTracker for 5 minutes at 37°C. Cells were washed in PBS and fixed with 4% paraformaldehyde for 10 minutes.

### Immunocytochemistry

Briefly, after 15 minutes fixation in 4% paraformaldehyde, cells were permeabilized for 10 minutes with PBS containing 0.2% BSA and 0.1% TritonX-100. Then, cells were incubated for 10 minutes with NH_4_Cl in PBS to quench free aldehyde groups. Primary antibody was incubated overnight. After washing, cells were incubated with fluorochrome-conjugated secondary antibodies for 1 hour. Finally, slides were washed, incubated with the nuclear stain and mounted. Antibodies were as follow: antibody for the surface of intact mitochondria (1:300; MAB1273, Millipore); MCT-4 (1:100; Santa Cruz); secondary antibody for immunofluorescence was Alexa orange-red 546 nm (1:500; Invitrogen).

### Western blotting

Cells were harvested in lysis buffer (RIPA buffer) containing protease and phosphatase inhibitors and centrifuged at 13,000x g for 10 minutes at 4°C to remove insoluble debris. Protein concentrations were analyzed using Bradford assay (Biorad). 30 μg of proteins were loaded and separated by SDS-PAGE and transferred to a 0.2 μm nitrocellulose membrane (Fisher Scientific). After blocking for 1 hour in TBST (10 mM Tris-HCl pH 8.0, 150 mM NaCl, 0.5% Tween-20) with 5% non-fat dry milk, membranes were incubated with the primary antibody for 1 hour or overnight, washed and incubated for 1 hour with horseradish peroxidase-conjugated secondary antibodies. The membranes were washed and incubated with an enhanced chemi-luminescence substrate (ECL; Thermo Scientific). The antibodies were as follow: β-actin (1:5000; Sigma); PKM2 (1:200; Cell Signaling); OXPHOS (1:500; MitoSciences).

### Gene expression analysis

Gene expression was analyzed after 24 hours of culture by quantifying gene transcripts. RNA was extracted with NucleoSpin RNA II (Macherey-nagel, Düren, Germany) and the retrotranscription was performed with MuLV Reverse Transcriptase (Applied Biosystems, Foster City, Ca, USA). Real-Time Polymerization Chain Reaction (Real-time PCR) was performed by amplifying 1 μg of cDNA using the Light Cycler instrument and the Universal Probe Library system (Roche Applied Science, Monza, Italy). Probes and primers were selected using the web-based assay design software (ProbeFinder: http://www.roche-applied-science.com). The protocol of amplification was: 95°C for 10 minutes; 95°C for 10 seconds, 60°C for 30 seconds, and 72°C for 1 second for 45 cycles; 40°C for 30 seconds. β-actin was used as housekeeping gene to normalize the expression of the genes of interest [43]. The results were expressed as a ratio between gene of interest and β-actin gene. All experiments were performed in triplicates.

### Measurement of reactive oxygen species

5-(and 6-) carboxy-2’,7’-dichlorodihydrofluorescein diacetate (CM-H2DCFDA; C369) was from Invitrogen. CM-H2DCFDA is a cell-permeable molecule used to measure intracellular ROS. MSC were incubated with 10 μM CM-H2DCFDA for 5 minutes at 37°C, then washed with PBS and analyzed by flow cytometry. To control for staining specificity, cells were also treated with 10 mM of NAC overnight prior to CM-H2DCFDA incubation. The production of ROS by MSC challenged with the conditioned medium of OS cell line Saos-2 was evaluated by measuring fluorescence emission following the conversion of 2’,7’-dichlorofluorescin-diacetate (DCFH-DA) into DCF by ROS produced intracellularly. All samples were analyzed on a flow cytometer EPICS XL-MCL (Beckman Coulter, Fullerton, CA, USA). The acquisition gate was established based on forward and side scatter parameters, and each sample was run until 10,000 events were acquired in the gate analysis. The fluorescent signal generated by ROS production was recorded as mean channel of fluorescence intensity (MC), and the effect of the conditioned media was expressed as a ratio between “MC of activated MSC” and “MC of non-activated MSC”.

### L-lactate assay

Lactate was measured in the culture media with lactate assay kit (EnzyChrome, BioAssay Systems) according to the manifacturer's instruction.

### Animal studies

Animals were housed and maintained in a pathogen-free environment/barrier facility at the University of Modena and Reggio Emilia, Modena, Italy. 1×10^6^ of 143B human cells were subcutaneously injected with reduced growth factor matrigel (BD Biosciences) in five-week-old severe combined immunodeficient (SCID)-bg/bg male mice (Charles River Laboratories International). Mice were sacrificed after three weeks, and tumor samples were collected for histological analysis. The Animal Ethics Committee of Modena approved all the experimental protocols.

### Immunohistochemistry

Five-micron paraffin sections were stained with MCT-4 antibody. Sections were dewaxed, rehydrated through graded ethanol series, and antigen retrieval was performed with 10 mM citrate buffer, pH 6.0 for 15 minutes using a microwave. The cooled sections were blocked with 3% hydrogen peroxide and next incubated with 5% BSA for 1 hour, followed by primary antibodies overnight at 4°C. Biotinylated secondary antibodies followed by streptavidin-horseradish peroxidase conjugated were from Dako. Immunoreactivity was revealed with the DAB solution (Dako). Sections were counterstained with hematoxylin.

### ATP assay

Specific gene silencing was obtained by siRNA technology associated with electromicroporation technology. Cells were trypsinized at semi-confluence and counted. 100 microliters of cell suspension containing 2×10^6^ cells and 4 nmol of specific or control siRNA (Dharmacon) were transferred into a 1-mm cuvette, and an electrical field was applied to induce siRNA cellular internalization (MicroPorator MP-100; Digital BioTechnology). For the RNA isolation, after electroporation, Saos-2 cells were transferred into 2 mL of complete growth medium and seeded in 12-well plates (3×10^5^ cells per well). After 24 hours, cells were lysed and RNA was extracted as described above. *For ATP assay*, after electroporation, Saos-2 cells were transferred into 3 mL of complete growth medium and seeded in 6-well plates (8×10^5^ cells per well). For the analysis of ATP content, cells were lysed by using boiling ATP lysis buffer [0.1 M Tris(hydroxymethyl)aminomethane (Tris base) (Sigma) and 2 mM EDTA adjusted to pH 7.75 with acetic acid (Sigma) and 2.5% Dodecyltrimethylammonium bromide (Sigma)]. The ATP content was measured with the ATP determination kit (Molecular Probes). The luciferase signal was normalized with the Log of the total protein concentration measured by Bradford assay (Bio-Rad Laboratories). All experiments were performed in triplicates.

### Migration assay

Transwells with uncoated permeable support and 8 μm pores were used. 4×10^4^ viable Saos-2 cells were seeded per well and incubated in 5% CO_2_ at 37°C for 12 hours. All functional experiments were performed in triplicates.

### In vitro scratch assay

Cells were plated onto the 60-mm dish to create a confluent monolayer. Cell monolayers were scraped in a straight line to create a ‘‘scratch’’ with a p200 pipet tip. Dishes were placed in a tissue culture incubator at 37°C for 24 hours. The images acquired for each sample were photographed under a phase-contrast microscope. To obtain the same field during the image acquisition, markings were created as reference points close to the scratch. The rate of migration was measured by quantifying the number of cells that moved from the edge toward the center of the scratch. Image J software was used to measure the wound closure [44].

### Statistical analysis

Quantitative results were expressed as arithmetic mean plus or minus the standard error of the mean (SEM) from at least 3 independent experiments. Mann-Whitney test was performed as unpaired comparison for two independent variables. All p values <0.05 were considered as statistically significant. *t*-test was also applied when appropriate. The statistical analysis was performed by StatView5.01 software (SAS Institute Inc., Cary, NC). The changes in ROS production after exposure to conditioned media were evaluated by a nonparametric test for paired data (Wilcoxon signed rank test).
